# Unilateral lateral rectus recession is an effective surgery for intermittent exotropia in young children

**DOI:** 10.1186/s12886-020-01778-2

**Published:** 2021-01-06

**Authors:** Oriel Spierer, Abraham Spierer

**Affiliations:** 1 Assuta Hospital, Ramat HaHayal, Tel Aviv, Israel; 2 Pediatric Ophthalmology and Adult Strabismus Unit, E. Wolfson Medical Center, Holon, Israel; 3 Sackler Faculty of Medicine, Tel Aviv University, Tel Aviv, Israel; 4 Pediatric Ophthalmology and Adult Strabismus Unit, Sackler Faculty of Medicine, E. Wolfson Medical Center, Tel Aviv University, Tel Aviv, Israel; 5 Goldschleger Eye Institute, Sheba Medical Center, Tel Hashomer, Israel

**Keywords:** Unilateral lateral rectus recession, Exotropia, Moderate‐angle exotropia, Strabismus, Pediatric ophthalmology

## Abstract

**Background:**

Different surgical methods have been suggested for the correction of intermittent exotropia. Unilateral lateral rectus recession has been described as a surgical alternative for small and moderate-angle exotropia. In general, previous studies did not focus on the outcomes of unilateral lateral rectus recession in young children with intermittent exotropia. The purpose of this study is to evaluate the surgical outcomes of unilateral lateral rectus recession in the treatment of moderate-angle exotropia (≤ 25 PD (prism diopters)) in children.

**Methods:**

The charts of all patients younger than 12 years of age with moderate-angle exotropia (up to 25 PD) who were operated during the years 2006–2018 were retrospectively reviewed. Fifty-eight patients underwent unilateral lateral rectus recession and had a minimum follow up of 6 months. The angle of exotropia (PD) before and after surgery and the success rate were documented.

**Results:**

Mean age at surgery was 6.4 ± 1.9 (range 3.5–11.0) years. Exotropia improved from a preoperative angle of 21.4 ± 4.0 PD to 3.5 ± 5.9 PD postoperatively (*p* < 0.001). Success rate, defined as deviation of ≤ 10 PD, was achieved in 86.2%. There were 2 (3.4%) cases of overcorrection (consecutive esotropia). There were no intra- or postoperative complications. The mean follow-up duration after surgery was 2.3 ± 1.7 years.

**Conclusions:**

In children with moderate angle exotropia, good postoperative success rate was achieved by performing unilateral lateral rectus recession.

## Background

Intermittent exotropia is the most common type of exotropia. Different surgical methods have been suggested for the correction of intermittent exotropia. Bilateral lateral rectus recession and recession of the lateral rectus with resection of the medial rectus muscles in one eye are the most encountered methods [1]. While these types of surgery are the most abundant among strabismus surgeons, unilateral lateral rectus recession has been described. Its efficiency was disputed in the past and the indications for its performance are still controversial [2, 3].

Many investigators [4–7] agree that unilateral lateral rectus recession may be effective in exotropia of 15–20 PD, reporting surgical success rate ranging from 72–90% in these angles [4–11]. Others [8] suggest uni-muscle surgery in exotropia of 30 PD and lower. A recent study comparing between unilateral lateral rectus surgery and lateral rectus recession combined with medial rectus resection for the treatment of intermittent exotropia reported similar results for both surgical approaches [10]. Another study found both methods to be comparable 2 years after surgery, while after 3 years, the recurrence rates were lower in the lateral rectus recession combined with medial rectus resection group than in the unilateral lateral rectus surgery group [12]. Kim et al. [13] have reported contralateral lateral rectus recession to be a safe and effective procedure for the treatment of recurrent exotropia after recession of the lateral rectus with resection of the medial rectus for intermittent exotropia.

Operating on one muscle instead of two muscles has the advantages of shortening the anesthesia time and diminishing the risks associated with the surgery such as scleral perforation, retinal detachment and endophthalmitis. It also spares more muscles for future intervention if needed. Probably its major advantage is the lower rate of overcorrection and consecutive esotropia [4–8, 10].

A concern in unilateral lateral rectus recession is postoperative lateral incomitance. This concern was ruled out in some studies, [5–7] and in others it was verified only when the lateral rectus muscle was recessed more than 9 mm [6, 8].In our previous study [9] we have found that unilateral lateral rectus recession may be as effective as bilateral lateral rectus recession in the treatment of patients with exotropia. Being encouraged by our results, we have proceeded operating our patients with moderate-angle intermittent exotropia (up to 25 PD) with unilateral lateral rectus recession. In general, previous studies did not focus on the outcomes of unilateral lateral rectus recession in young children with intermittent exotropia. The purpose of this study is to evaluate the surgical outcomes obtained by unilateral lateral rectus recession for the correction of moderate-angle intermittent exotropia (up to 25 PD) in young children.

## Methods

The clinical charts of consecutive patients with intermittent exotropia of up to 25 PD who were operated between January 2006 and October 2018 were reviewed. Exclusion criteria included paralytic strabismus, restrictive strabismus, convergence insufficiency exotropia, deep amblyopia, a neurological deficit or previous ocular surgery. Patients with follow up of less than 6 months after the surgery were also excluded. In our practice, in patients age 12 years or older, the adjustable suture technique is routinely done. Due to poor cooperation, this technique is not suitable for younger children, thus patients in whom the adjustable suture technique was used are not included in this study.

Data collection and analysis included: Age at onset of the deviation, age at surgery, the angle of exotropia (in PD), the amount of the lateral rectus muscle recession (in mm), the amount of strabismus correction achieved by 1 mm of lateral rectus recession, the rate of overcorrection and the success rate (which was defined as exodeviation of ≤ 10 PD or esodeviation of ≤ 5 PD on last follow-up). Cases of lateral incomitance (if any) were documented.

The prism cover test was used to measure the angle of exotropia for both distance (6 meters) and near (30 centimeters) fixation. Titmus Fly Test was used to test stereopsis in cooperative subjects.

Informed consent was obtained from a parent and/or legal guardian of all subjects prior to surgery. This study was approved by the Institutional Review Board of Assuta Hospital, Ramat HaHayal, Tel Aviv, Israel, and complied with the principles outlined in the Declaration of Helsinki.

### Surgical technique

Under general anesthesia a fornix incision was done, and a lateral rectus recession was performed. A 6 − 0 Vicryl suture was placed 1 mm from the muscle insertion. The muscle was disinserted and then sutured to the sclera at its new location, measured from the original muscle insertion. Angle of deviation of up to 15 PD was corrected by a 7 mm lateral rectus recession, 16–20 PD angle of deviation was corrected by an 8 mm recession, and 21–25 PD was corrected by a 9 mm recession.

### Statistical analysis

To compare between the preoperative and postoperative angle of deviation for distance and near, the paired samples t-test was used. Analyses were two-tailed, and significance was set at 5%. Statistical analysis was performed with SPSS™ program. Data are presented as mean ± standard deviation.

## Resuts

Fifty-eight consecutive patients with intermittent exotropia comprised the study population with a mean postoperative follow-up of 2.3 ± 1.7 (range 0.5–9.0) years. Patient demographics are summarized in Table 1. In all patients, anterior segment examination and fundoscopy were unremarkable.

**Table 1:** Preoperative and Postoperative Date for Children with Moderate-Angle Exotropia Undergoing Unilateral Lateral Rectus Recession (*n* = 58)

	Mean ± SD	Range
Age at Onset (y)	2.7 ± 1.8	0.5–8.0
Age at Surgery (y)	6.4 ± 1.9	3.5–11.0
Angle of Exotropia (prism diopters)		
Preoperative Angle, Distance	21.4 ± 4.0	14–25
Preoperative Angle, Near	9.2 ± 7.2	0–25
Postoperative Angle, Distance	3.5 ± 5.9	14 ET to 25 XT
Postoperative Angle, Near	1.6 ± 3.3	14 ET to 12 XT

*ET esotropia; XT exotropia*

Age at onset of deviation was 2.7 ± 1.8 (range 0.5–8.0) years. Visual acuity in both eyes ranged between 20/20 and 20/40 in all patients with a mean refractive error of -0.4 ± 2.5 (range − 11.0 to + 4.0) D. Age at surgery was 6.4 ± 1.9 (range 3.5–11.0) years. The mean interval between the onset of the strabismus and surgery was 3.7 ± 1.8 (range 1.0–8.5) years.

Preoperative mean angle of deviation was 21.4 ± 4.0 (range 14.0–25.0) PD for distance and 9.2 ± 7.2 (range 0–25.0) PD for near fixation. At last follow-up examination the mean postoperative angle of deviation was 3.5 ± 5.9 PD (range 14.0 PD esotropia to 25.0 PD exotropia) at distance (p < 0.001) and 1.6 ± 3.3 PD (range 14.0 PD esotropia to 12.0 PD exotropia) at near (p < 0.001). Compared to pre-operative examination the angle of deviation was changed by an average of 18.5 ± 7.3 PD at distance and 7.9 ± 7.7 PD at near at last follow-up.

Success rate (defined as deviation of ≤ 10 PD, at last follow-up examination) was 86.2% with 50 patients having a desired outcome. Six (10.3%) patients had exotropia of > 10 PD (12–25 PD of exotropia) on last follow up examination. Three of these patients underwent a second surgery of lateral rectus recession in the second eye. Two (3.4%) patients had consecutive esotropia of > 5 PD. One of the patients underwent unilateral medial rectus recession 9 months after the first surgery. The second patient was lost to follow-up 6 months postoperatively.

The mean amount of lateral rectus recession performed was 8.4 ± 0.5 (range 7.0–9.0) mm and was planned for each patient according to the angle of deviation for distance. The amount of correction achieved by 1 mm of recession of the lateral rectus was calculated for each muscle. A mean correction of 2.2 ± 0.9 PD per 1 mm of recession of the lateral rectus was found. Ten (17.2%) patients underwent in addition inferior oblique recession for inferior oblique overaction.

Following surgery, binocular vision was found to be full in 43 (74.1%) patients, partial stereopsis was found in 10 (17.2%) and absent stereopsis in 2 (3.5%) patients. In 3 (5.2%) patients the test could not have been performed due to lack of patient cooperation.

No intra- or postoperative complications were encountered in any of the patients. No cases of lateral incomitance were observed.

## Discussion

Our study found that unilateral lateral rectus recession is effective in treating moderate-angle intermittent exotropia in young children. A high success rate of 86.2% was achieved with a mean postoperative angle of deviation of 3.5 PD for distance and 1.6 PD for near.

The success rate of unilateral rectus recession for intermittent exotropia differs among studies. Suh et al. [10] compared the surgical outcomes of patients who underwent unilateral lateral rectus recession and patients who underwent lateral rectus recession and medial rectus resection for exotropia of 20–25 PD. The surgical success rate did not differ significantly between groups at last follow-up, with 45.9% in the lateral rectus group and 39.4% in the recession-resection group. Another report found a surgical success rate of 60.9% at an angle of 25 PD [14].We have previously reported a success rate of 69% in unilateral lateral rectus recession in moderate-angle exotropia [9]. That study, as most studies in this era, included patients with diversity of ages: children, teenagers, and adults. In the present study, we included only young children (whom their surgical outcomes were not reported in the past) to investigate whether the success rate would be similar. We found a high success rate of 86.2% in the population of young children, higher than most papers published. It is unclear whether young children respond better to unilateral rectus recession than older children and adults. Differences in success rates among studies might be due to different follow-up periods, demographic variances, like age and surgeon experience.

Among the advantages of operating on 1 muscle instead of 2 muscles are shortening the general anesthesia time and diminishing the ocular risks associated with surgery on 2 muscles. Bearing in mind that the rate of residual and recurrent exotropia after the first surgery is not low, sparing of other horizontal muscle for future surgery is another positive thing to consider. Nevertheless, unilateral lateral rectus recession major advantage is the low rate of overcorrection. When overcorrection after surgery for intermittent exotropia occurs, it may result in a constant esotropia with debilitating diplopia and loss of stereopsis, necessitating another surgery of lateral rectus advancement or medial rectus recession. Suh and colleagues [10] reported that postoperative overcorrection is less common in unilateral lateral rectus surgery. While in the recession-resection group, 9% of the patients showed overcorrection at final visit, no patient showed an overcorrection after surgery in the unilateral lateral rectus group [10]. In the present study there were 2 cases (3.4%) of consecutive esotropia.

Our surgical approach was the same for a true divergence excess as well as for simulated divergence excess exotropia. In both cases, we performed lateral rectus recession, in an amount based on the distance angle of deviation. For this reason, we do not routinely perform a patch test in cases where the angle of deviation for distance is greater than that for near.

Performing a unilateral surgery for exodeviation of 25 PD would necessitates a large recession of the lateral rectus muscle – between 8 and 9 mm. The common recommendation in previous published studies was to perform unilateral lateral rectus surgery only in smaller angles of strabismus. Some stated that if a recession of more than 7 mm is needed, then a bilateral surgery should be done [2]. However, others performed unilateral surgery with as large recession of the muscle as 11.5–12 mm [4]. Lateral incomitance is a possible complication of large recession done unilaterally. This complication was found to be transient and uncommon [4, 5]. It is possible that lateral incomitance is more prevalent when the recession of the lateral rectus muscle is more than 8 mm [8]. In our study, no lateral incomitance was observed in any of the patients, even in those in which the muscle was recessed by 9 mm. We believe that the risk for lateral incomitance should not prevent surgeons from performing lateral rectus recession of up to 9 mm, as this complication is probably rare. It is possible that lateral incomitance occurs if there is a muscle slippage after surgery, especially if in addition there is underaction of the lateral rectus.

The retrospective nature of our study is its main limitation. As in some patients follow up was only 6 months, longer follow-up may reveal lower success rate. This limitation is common to all studies in this era. The main strengths of the study are the homogeneity of patients and the relatively long mean follow-up period.

## Conclusions

Unilateral lateral rectus recession is an effective surgery for treating moderate angle of intermittent exotropia (up to 25 PD) in young children. The success rate was higher than most studies in this era, especially in exotropia angles of 25 PD. It is unclear how age affects surgical results. Randomized controlled trials comparing lateral rectus recession and other surgical approaches among different ages are needed.

## Data Availability

The datasets used and/or analysed during the current study are available from the corresponding author on reasonable request.

## Abbreviations

ET: Esotropia
PD: Prism diopters
XT: Exotropia
